# HMF-DEIM: High-Fidelity Multi-Domain Fusion Transformer for UAV Small Object Detection

**DOI:** 10.3390/s26072187

**Published:** 2026-04-01

**Authors:** Lan Ma, Yun Luo, Jiajun Xu

**Affiliations:** College of International Studies, National University of Defense Technology, Nanjing 210012, China

**Keywords:** UAV imagery, small object detection, end-to-end transformer detection, multi-scale feature fusion, real-time detection, hierarchical feature differentiation, cross-scale contextual aggregation

## Abstract

Unmanned aerial vehicle (UAV) small object detection faces critical challenges including irreversible geometric detail loss during multi-level downsampling, cross-scale feature distortion from interpolation blur and aliasing, and limited long-range dependency modeling due to constrained receptive fields. To address these limitations, we propose HMF-DEIM (High-Fidelity Multi-Domain Fusion Transformer for UAV Small Object Detection), an end-to-end architecture tailored for UAV small object detection. First, we design a lightweight hierarchical differentiation backbone that removes redundant deepest-layer features (P5) to prevent tiny object information loss, employing Multi-Domain Feature Blending (MDFB) in shallow layers for geometric detail preservation and a Hierarchical Attention-guided Feature Modulation Block (HAFMB) in deep layers for global semantic modeling. Second, we develop a full-chain high-fidelity feature transformation framework comprising Channel-Adaptive Shift Upsampling (CASU) for interpolation-free resolution recovery, Multi-scale Context Alignment Fusion (MCAF) for bridging deep–shallow semantic gaps via bidirectional gating, and Diversified Residual Frequency-aware Downsampling (DRFD) for aliasing suppression through a three-branch parallel architecture. Finally, we devise the FocusFeature module that aligns multi-scale features to a unified scale and employs parallel multi-scale large-kernel depthwise convolutions to capture cross-scale long-range dependencies, generating dual-scale (P3/P4) features balancing details and semantics. Experiments demonstrate that HMF-DEIM outperforms DEIM on VisDrone2019 test by 0.405 mAP^50^ (+2.1%) and 0.235 mAP^50–95^ (+1.6%), with a remarkable 21.3% relative improvement in AP_*s*_ for tiny objects, while maintaining real-time inference (465 FPS with TensorRT FP16) on an NVIDIA A100 GPU with only 11.87M parameters and 34.1 GFLOPs. Further validation on AI-TOD v2 and DOTA v1.5 datasets confirms robust generalization across diverse aerial scenarios, making it a practical solution for resource-constrained UAV applications.

## 1. Introduction

Unmanned Aerial Vehicles (UAVs) have evolved from simple flight platforms into intelligent systems equipped with onboard edge computing capabilities. These systems can process complex algorithms in real time without continuous ground station communication, enabling critical applications such as traffic monitoring, disaster relief, tactical reconnaissance, and automated surveillance [1,2,3,4]. However, UAV vision systems face severe challenges in dense urban environments as follows: object detection must simultaneously achieve high accuracy and real-time performance. The unique characteristics of aerial imagery—including extreme scale variations, complex backgrounds, and diverse viewpoints—pose fundamental technical barriers to conventional computer vision frameworks.

In complex UAV aerial scenarios, small object detection faces multiple technical challenges. Traditional convolutional neural networks often cause irreversible loss of small object geometric details during multiple downsampling operations, while fixed receptive field designs cannot simultaneously accommodate feature extraction for objects of different scales [5]. Although attention mechanisms show potential in global modeling, their effectiveness in shallow network layers is limited [6,7], and conventional feature maps often carry significant redundant information, leading to higher computational overhead. Particularly, the P5 layer (stride 32) of standard backbone networks causes complete loss of tiny object information [8]. **To address this feature degradation problem**, we design a lightweight backbone network specifically tailored for UAV scenarios, adopting a **hierarchical differentiation strategy** and **removing redundant deepest-layer features**. In shallow layers (P2/P3), the Multi-Domain Feature Blending (MDFB) module maximizes feature reuse through dense connections and split–merge strategies; in deep layers (P4), the Hierarchical Attention-guided Feature Modulation Block (HAFMB) employs channel splitting strategies, utilizing single-head self-attention mechanisms to enhance global semantic modeling.

Meanwhile, due to dramatic variations in flight altitude and viewing angles, objects of the same category may exhibit vastly different scale distributions [9,10], posing severe challenges for **cross-scale feature transformation**. Existing feature pyramids employ simple interpolation methods during upsampling stages, which cannot reconstruct sub-pixel level details; during downsampling stages, aliasing effects caused by strided convolutions convert high-frequency details into low-frequency noise [11,12]. To address this information distortion problem, we propose a high-fidelity feature transformation framework encompassing both upsampling and downsampling processes. In the upsampling stage, Channel-Adaptive Shift Upsampling (CASU), employs a channel cyclic shift mechanism to avoid interpolation blur; subsequently, the Multi-scale Context Alignment Fusion (MCAF) module utilizes bidirectional gating mechanisms to dynamically coordinate deep and shallow layer features, ensuring semantic alignment; in the downsampling stage, Diversified Residual Frequency-aware Downsampling (DRFD) **adopts a three-branch parallel architecture** to suppress high-frequency aliasing effects from multiple dimensions.

More critically, the effective receptive field of traditional convolutional networks is far smaller than the theoretical value, lacking sufficient long-range dependency modeling capability for tiny objects [5,13,14], which constitutes a **fundamental bottleneck for contextual reasoning**. To overcome this limitation, we develop the FocusFeature module as the core aggregation node. This module **first aligns multi-scale features to a unified scale**, and it then employs **parallel multi-scale large-kernel depthwise convolutions** to significantly expand the effective receptive field, collaboratively capturing cross-scale long-range dependencies. The module ultimately generates semantically rich P3 and P4 features. This **dual-scale feature generation strategy (P3/P4)** fully leverages the high-resolution advantage of P3 and the semantic abstraction capability of P4, significantly reducing decoder computational complexity while maintaining detection accuracy.

From the perspective of detection framework evolution, traditional methods are mainly divided into CNN-based single-stage detectors (such as RetinaNet [15] and the YOLO series [16,17,18]) and two-stage detectors (such as Faster R-CNN [19]). Although these CNN methods perform well in general scenarios, they face significant challenges in UAV small object detection. In recent years, Transformer-based detectors have demonstrated new possibilities. DETR [20] redefined object detection as a set prediction problem by introducing self-attention mechanisms [21], eliminating traditional post-processing steps. RT-DETR [22] achieved real-time detection on this foundation. Recently, DEIM [23] achieved significant breakthroughs based on the DETR framework. By introducing the Dense O2O matching strategy and Matchability-Aware Loss, DEIM effectively addresses the sparse supervision problem in traditional one-to-one matching, demonstrating excellent performance on general object detection tasks. Nevertheless, regardless of whether they employ CNN or Transformer architectures, most current detectors are tailored for general-purpose applications and fail to effectively handle the distinctive challenges posed by aerial imagery, such as extreme scale variations, densely packed objects, and complex viewpoint changes.

Based on DEIM’s excellent performance in general object detection and its end-to-end training advantages, we select it as our baseline model. However, DEIM still faces numerous challenges in UAV aerial scenarios, as follows: its standard backbone network has insufficient feature representation capability when processing multi-scale small objects, the original feature fusion mechanism struggles to effectively integrate semantic and detail features from different hierarchical levels, and traditional downsampling operations easily cause loss of small object features. To resolve these problems, we propose HMF-DEIM (Hierarchical Multi-scale Fidelity Transformer), an end-to-end detection architecture specifically tailored for UAV small object detection. The core design philosophy of this architecture is reflected in the following aspects:

**Hierarchical differentiation in the feature extraction stage**: The lightweight backbone network removes redundant deepest-layer features, adopting a hierarchical strategy of MDFB (shallow layers) and HAFMB (deep layers), achieving progressive feature extraction from geometric details to semantic abstraction while maintaining parameter efficiency.

**Full-chain fidelity in the feature transformation stage**: The CASU-MCAF-DRFD framework constitutes a complete bidirectional closed loop—CASU achieves lossless upsampling through cyclic shifting, MCAF resolves the semantic gap between deep and shallow layers through gating mechanisms, and DRFD suppresses aliasing noise through a three-branch architecture, ensuring information integrity during cross-scale transfer.

**Cross-scale reasoning in the feature aggregation stage**: The FocusFeature module breaks through the locality limitation of convolutional networks through spatial alignment combined with parallel large-kernel convolutions, enabling the model to establish macro-level contextual associations while preserving micro-level details, and generates dual-level features balancing details and semantics for final decoding.

In summary, our main contributions are as follows:

1. **Hierarchical differentiation backbone network design**: We propose a lightweight backbone network specifically tailored for UAV scenarios, adopting a **hierarchical differentiation strategy** and **removing redundant deepest-layer features** to prevent small object information loss. In the shallow stage, MDFB maximizes feature reuse through dense connections and split–merge strategies, ensuring high-fidelity preservation of small object geometric features; in the deep stage, HAFMB employs a channel splitting strategy, utilizing single-head self-attention for global semantic modeling on partial channels, suppressing background noise while maintaining lightweight characteristics.
2. **Full-chain high-fidelity feature transformation framework**: We propose a high-fidelity feature transformation framework encompassing both upsampling and downsampling processes. In the upsampling stage, CASU employs a channel cyclic shift mechanism to avoid interpolation blur, effectively reconstructing sub-pixel level details; in the fusion stage, MCAF utilizes bidirectional gating mechanisms to achieve semantic alignment between deep and shallow layer features; in the downsampling stage, DRFD suppresses high-frequency aliasing effects, ensuring full-chain feature flow fidelity.
3. **Multi-scale context-focused aggregation and dual-scale feature generation**: We develop the FocusFeature module as the core aggregation node, breaking through the locality limitation of convolutional neural networks. This module **first aligns multi-scale features to a unified medium resolution**, and it then employs parallel multi-scale large-kernel depthwise convolutions to expand the effective receptive field, capturing cross-scale long-range dependencies, generating semantically rich medium-resolution features, and producing deep-layer features through DRFD downsampling, reducing decoder computational complexity while maintaining high-resolution detail perception.
4. **End-to-end UAV detection architecture**: We propose the HMF-DEIM architecture specifically suited for UAV small object detection. By removing deepest-layer features that cause tiny object information loss and by utilizing a dual-scale decoding strategy to optimize fine-grained feature distribution, we achieve an optimal trade-off between detection accuracy and inference speed. Experiments demonstrate that HMF-DEIM significantly outperforms the baseline DEIM and other state-of-the-art detectors on aerial datasets including VisDrone2019, AI-TOD-v2, and DOTA-v1.5, validating the effectiveness and generalization capability of our method.

The remainder of this paper is organized as follows: Section 2 comprehensively reviews related work, Section 3 details our proposed method, Section 4 presents experimental results, and Section 5 concludes the paper.

## 2. Related Work

### 2.1. Transformer-Based Detection Frameworks

DETR [20], proposed by Carion et al., redefined object detection as a set prediction problem, pioneering the application of Transformers in object detection and eliminating the need for hand-designed components such as RPN and NMS. DETR employs the Hungarian algorithm to achieve one-to-one (O2O) matching, assigning a unique prediction box to each object and enabling end-to-end training. However, DETR exhibits notable deficiencies in convergence speed and computational complexity. To address these issues, researchers have proposed various improvements. Deformable DETR [24] introduced deformable attention mechanisms, improving convergence efficiency and multi-scale feature processing capability through sparse spatial sampling. DN-DETR [25] accelerated training convergence through query denoising strategies, while DINO [26] further improved performance by combining improved denoising anchor boxes with contrastive denoising training. DAB-DETR [27] enhanced localization capability through dynamic anchor boxes, and Efficient DETR [28] optimized decoder initialization using dense query selection mechanisms.

Although these methods have achieved significant accuracy improvements, the increased computational complexity brought by stacked encoder–decoder architectures or additional auxiliary modules limits their real-time deployment. RT-DETR [22] first achieved real-time object detection based on the DETR framework, applying the Transformer encoder only on the deepest-layer features and integrating multi-scale information through efficient feature fusion strategies, significantly reducing computational overhead while maintaining end-to-end advantages. RT-DETRv2 [29] further adopted IoU-aware query selection strategies and improved loss functions. D-FINE [30] redefined the regression task as a fine-grained distribution optimization problem, combined with knowledge distillation techniques, surpassing YOLO series models in real-time performance.

Recently, DEIM [23] effectively addressed the sparse supervision and low-quality matching problems in O2O matching through a Dense O2O matching strategy and Matchability-Aware Loss (MAL), significantly accelerating model convergence and achieving excellent performance on general object detection tasks. However, DEIM still faces the following challenges in UAV aerial scenarios: its standard backbone network has insufficient feature representation capability when processing multi-scale objects, leading to higher miss rates for small objects; the original feature fusion mechanism has limitations in cross-scale information aggregation; and traditional downsampling operations easily cause loss of small object features. Based on these observations, we select DEIM as our baseline model and optimize the backbone network architecture, feature fusion mechanism, and cross-scale feature aggregation strategy, thereby achieving a better accuracy–speed trade-off.

### 2.2. Object Detection in UAV Images

Due to the nature of UAV flight, capturing images from drones presents numerous difficulties, including cluttered backgrounds, variations in viewing angles, changes in altitude, shifting lighting conditions, blur caused by motion, and partial occlusion of targets. Furthermore, the dramatic scale variations and high proportion of small objects in aerial scenarios impose higher requirements on detectors’ multi-scale feature extraction capabilities. To address these challenges, researchers have proposed solutions from the following three directions: **backbone network design, feature fusion mechanisms, and downsampling strategies**, corresponding to the three major challenges addressed in this paper, as follows: **feature degradation, cross-scale distortion, and contextual bottlenecks**.

**In backbone network design**, CenterNet [31] adopted a lightweight Hourglass network to preserve spatial details, CSPNet [32] enhanced feature reuse through cross-stage partial connections, and EfficientNet [33] balanced network depth, width, and resolution through compound scaling. These works demonstrate that lightweight backbones need to preserve fine-grained geometric information in shallow layers, enhance semantic abstraction in deep layers, and reduce parameter redundancy through feature reuse. CSPNet’s cross-stage connections inspired MDFB’s feature split–merge strategy, and EfficientNet’s compound scaling inspired HAFMB’s channel splitting strategy. Additionally, this paper removes deepest-layer features to focus on high-resolution modeling at the P2/P3/P4 three layers, addressing the problem of tiny objects vanishing at large-stride levels.

**In feature fusion mechanisms**, BiFPN [34] achieved multi-scale feature fusion through weighted bidirectional connections, ASFF [35] introduced adaptive spatial feature fusion by dynamically learning fusion weights, and NAS-FPN [36] discovered optimal feature pyramid structures using neural architecture search. These studies demonstrate that deep and shallow layer features require semantic alignment and adaptive fusion. However, existing methods mostly focus on learning fusion weights while ignoring upsampling detail loss and the deep–shallow layer semantic gap. ASFF’s adaptive fusion inspired MCAF’s gating mechanism, and BiFPN’s bidirectional connections inspired MCAF’s bidirectional interaction strategy. The CASU module in this paper transforms upsampling into spatial reorganization through channel cyclic shifting, fundamentally solving the interpolation blur problem.

**In downsampling and information preservation**, SPP [37] preserved multi-scale information through spatial pyramid pooling, CARAFE [38] proposed content-aware upsampling operators, and PConv [39] reduced redundant computation using partial convolutions. Zhang et al. [11] pointed out that traditional strided convolutions violate the sampling theorem and proposed anti-aliasing downsampling strategies. These works demonstrate that addressing feature aliasing from a frequency domain perspective is crucial for preserving high-frequency details. However, existing methods mostly adopt single downsampling strategies, struggling to simultaneously preserve geometric details and saliency features. The frequency domain analysis of anti-aliasing downsampling inspired DRFD’s three-branch architecture, which collaboratively maintains information integrity across the frequency domain, spatial domain, and saliency dimensions.

**In recent advances**, several works have continued to push the boundary of UAV small object detection. SMA-YOLO [40] improved small object sensitivity through sparse attention and multi-branch FPN design, BGF-YOLOv10 [41] introduced a lightweight architecture with an additional small object detection head for UAV perspectives, and FECI-RTDETR [42] explored lightweight RT-DETR variants for UAV infrared scenarios. Meanwhile, State Space Models (SSMs) have emerged as an alternative paradigm offering linear complexity with global modeling capability. Building upon the foundational S4 [43] and Mamba [44] architectures, Vision Mamba [45] and VMamba [46] successfully adapted SSMs for visual representation learning. In the detection domain, Mamba YOLO [47] proposed an SSM-based backbone for real-time detection. For UAV scenarios specifically, YOLOv5_mamba [48], YOLO-Mamba [49], HRMamba-YOLO [50], MV-YOLO [51], and Super Mamba [52] have explored integrating Mamba modules into YOLO-based frameworks for aerial and infrared imagery, while RemoteDet-Mamba [53] and MSDF-Mamba [54] investigated Mamba-based fusion for multi-modal aerial detection. However, most of these approaches simply insert SSM modules into existing backbones or necks without holistically redesigning the detection pipeline, and none explicitly addresses the deepest-layer feature redundancy problem that causes tiny object information loss in UAV scenarios.

Existing methods still have critical issues in UAV small object detection. General backbones do not consider the specificity of object scales in UAV scenarios, lacking fine-grained feature capture capability for small objects in shallow layers, while deepest layers cause tiny object disappearance. Traditional FPN/PAN adopts simple addition or concatenation, struggling to model cross-level semantic associations, with upsampling interpolation causing detail blur. Standard downsampling has limited spatial position preservation capability, easily losing small object boundary and texture information. Moreover, simple feature concatenation lacks spatial alignment and cross-scale contextual modeling.

In summary, existing research in lightweight backbone design, semantic-aligned fusion, and frequency-aware downsampling has laid the theoretical foundation for the HMF-DEIM architecture proposed in this paper. This paper addresses the above issues through a series of innovations as follows: the MDFB/HAFMB backbone network achieves hierarchical differentiation design while removing deepest-layer features to avoid tiny object information loss; the collaborative design of CASU and MCAF modules addresses upsampling detail loss and deep–shallow layer semantic gap problems; the DRFD three-branch architecture performs parallel processing across the frequency fidelity, spatial learning, and saliency preservation dimensions; and the FocusFeature module achieves cross-scale contextual reasoning through spatial alignment and parallel large-kernel convolutions.

## 3. Proposed Method

### 3.1. Overall Framework

**Figure 1:**
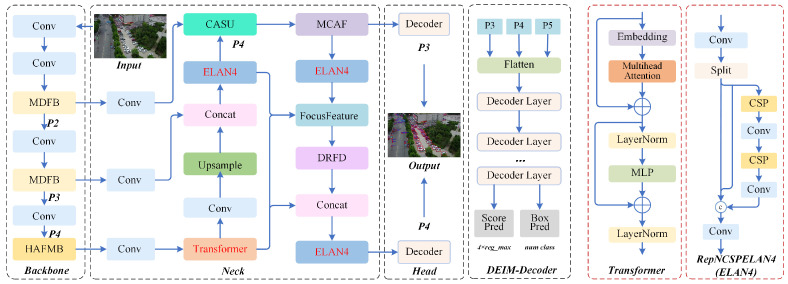
**Overall architecture of HMF-DEIM**. The model consists of four main components: a **Backbone** for multi-scale feature extraction (including CASU, RepNCSPELAN4 integrated with MDFB, CAV, and Transformer modules), a **Decoder** with embedding, multi-head attention, and prediction heads for score and box outputs, a **DEIM-Decoder** that further refines detection results through transformer layers.

Tiny object detection in UAV aerial imagery faces the dual challenges of extreme scale variations and background interference. Multiple downsampling operations in traditional convolutional networks cause irreversible loss of high-frequency details, while existing feature pyramids struggle to balance deep-layer semantics with shallow-layer geometric details when fusing multi-scale information, often introducing noise due to aliasing effects. To resolve these problems, we propose the HMF-DEIM architecture, as illustrated in Figure 1. This framework achieves optimization through the following four key designs: a lightweight backbone network based on MDFB and HAFMB, high-fidelity feature transformation covering the entire upsampling and downsampling process, cross-scale contextual aggregation based on multi-granularity large-kernel convolutions, and a decoder optimized for dual-scale features.

The overall pipeline is divided into four stages as follows: feature extraction, hybrid encoding enhancement, cross-scale focused aggregation, and distribution-aware decoding. In the feature extraction stage, we design a customized lightweight backbone network that adopts a hierarchical differentiation strategy and removes the redundant deepest layer P5. After initial convolution, the input image progressively generates the following three hierarchical features: P2 at the shallow layer with 4× downsampling, P3 at the middle layer with 8× downsampling, and P4 at the deep layer with 16× downsampling. In the shallow and middle layers, the Multi-Domain Feature Blending module is introduced to maximize inter-level feature reuse through split–merge strategies and dense connections, preventing the loss of tiny object geometric information. In the deep layer, the Hierarchical Attention-guided Feature Modulation Block is integrated, employing a channel splitting strategy where partial channels utilize self-attention for global semantic modeling while remaining channels preserve detail information, simultaneously introducing multi-scale depthwise convolutions to enhance local representation. The backbone network outputs the following three hierarchical features: $F_{P2}^{backbone}$, $F_{P3}^{backbone}$, and $F_{P4}^{backbone}$.

In the encoding and feature enhancement stage, the Transformer encoder is applied only to $F_{P4}^{backbone}$ for global contextual modeling. The encoded $F_{P4}^{enc}$ progressively recovers spatial resolution through a top-down pathway. First, $F_{P4}^{enc}$ is processed through Channel-Adaptive Shift Upsampling and added to $F_{P3}^{backbone}$ to obtain $F_{P3}^{fused}$. Subsequently, $F_{P3}^{fused}$ continues through Channel-Adaptive Shift Upsampling to the shallow-layer scale and undergoes gated fusion with $F_{P2}^{backbone}$, dynamically coordinating fine-grained features with deep-layer semantics to resolve the semantic gap problem, generating $F_{P2}^{final}$.

In the feature aggregation stage, three feature streams are fed into the FocusFeature module as follows: $F_{P2}^{final}$ carrying high-resolution details, $F_{P3}^{fused}$ representing middle layer features, and $F_{P4}^{enc}$ encoding semantic abstraction. This module first spatially aligns the three feature streams to the P3 scale. Specifically, $F_{P2}^{final}$ is reduced through frequency-aware downsampling, $F_{P4}^{enc}$ is enlarged through Channel-Adaptive Shift Upsampling, and $F_{P3}^{fused}$ undergoes direct channel alignment. The aligned features are concatenated along the channel dimension, and parallel large-kernel depthwise convolutions with kernel sizes of 5, 7, 9, and 11 are employed to expand the effective receptive field, collaboratively capturing cross-scale long-range dependencies and outputting $F_{P3}^{focus}$. To accommodate dual-scale decoding requirements, $F_{P3}^{focus}$ is further processed through frequency-aware downsampling to generate $F_{P4}^{focus}$, ensuring semantic alignment while suppressing aliasing noise.

In the decoding stage, the deeply optimized $F_{P3}^{focus}$ and $F_{P4}^{focus}$ are fed into the D-FINE Transformer decoder. The dual-scale strategy offers several advantages. Removing P5 prevents complete loss of tiny object information. P3 provides fine localization capability while P4 provides semantic context, with both being complementary. Compared to three-scale schemes, computational complexity is significantly reduced to meet real-time requirements.

### 3.2. Lightweight Backbone Network

Standard backbone networks employ fixed receptive field convolutions, making it difficult to simultaneously accommodate shallow-layer geometric information and deep-layer semantic abstraction, while dense convolution stacking introduces redundant parameters. In particular, the P5 layer with 32× downsampling causes tiny objects to have representations smaller than one pixel in feature maps, resulting in complete information loss. For example, a small object of 16 × 16 pixels corresponds to only 0.5 × 0.5 pixels at the P5 layer, where neither geometric nor semantic information can be preserved. Therefore, we remove the P5 layer and retain the following three hierarchical features: P2 at the shallow layer with 4× downsampling, P3 at the middle layer with 8× downsampling, and P4 at the deep layer with 16× downsampling.

**Figure 2:**
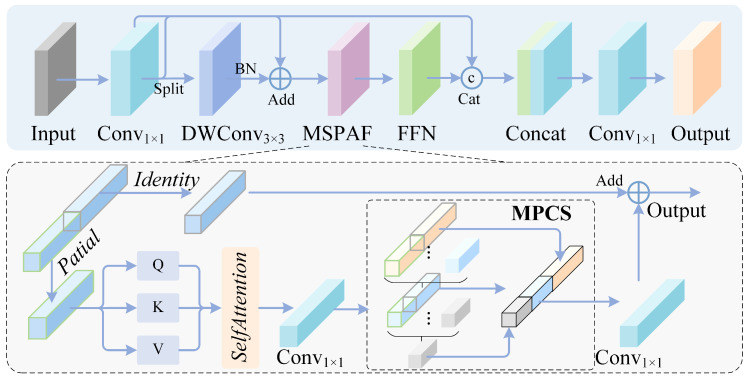
**Architecture of HAFMB.** Top is the main pipeline with ${\mathrm{Conv}}_{1\times 1}$, ${\mathrm{DWConv}}_{3\times 3}$, MSPAF, FFN, and feature concatenation. Bottom is detailed structure of MSPAF, including partial self-attention, identity mapping, and MPCS module with residual connection.

Our designed multi-stage backbone network adopts a hierarchical differentiation strategy. MDFB is introduced in shallow and middle layers to efficiently reuse multi-scale features and preserve geometric details, while HAFMB is deployed in the deep layer to enhance global semantic modeling, as illustrated in Figure 2. This hierarchical design fully accommodates the level-specific requirements where shallow and middle layers demand fine details while deep layers require semantic abstraction, maintaining parameter efficiency through feature reuse and channel splitting.

#### 3.2.1. Shallow and Middle Layer Feature Extraction Module

In shallow and middle layer stages, geometric details such as the edge textures of small objects are distributed across feature maps. The MDFB module achieves multi-scale feature interaction through a feature split–merge mechanism. Given input $X\in R^{C\times H\times W}$, it is first mapped to a higher dimension through 1 × 1 convolution, yielding $Y\in R^{C^{\prime }\times H\times W}$, where $C^{\prime }=2C$ doubles the channel capacity to provide sufficient representational bandwidth for the subsequent multi-branch processing. Subsequently, Y is decomposed into four parallel branches, each operating on the full or partial channels of Y to capture features at different granularities as follows:

(1) $$Y_{0}=\phi_{conv}^{\left(1\right)}\left(Y\right),\;Y_{1}=\phi_{DW}^{3\times 3}\left(\phi_{conv}^{\left(2\right)}\left(Y\right)\right),\;Y_{2},Y_{3}=\mathrm{Split}\left(Y\right)$$

where $\phi_{conv}$ denotes standard convolution and $\phi_{DW}^{k\times k}$ denotes depthwise convolution with kernel size *k* that performs spatial convolution independently per channel. The four branches serve distinct and complementary roles. Branch $Y_{0}\in R^{C^{\prime }/4\times H\times W}$ applies a standard 1 × 1 convolution to perform cross-channel feature recombination. Branch $Y_{1}\in R^{C^{\prime }/4\times H\times W}$ sequentially applies a 1 × 1 convolution followed by a 3 × 3 depthwise convolution, extracting local spatial patterns such as edges and textures while maintaining per-channel independence. Branches $Y_{2}$ and $Y_{3}$, each $\in R^{C^{\prime }/4\times H\times W}$, are obtained by evenly splitting Y along the channel dimension, where $Y_{3}$ directly preserves original low-level geometric information as an identity shortcut, while $Y_{2}$ is forwarded for further hierarchical refinement.

To enhance representational capability, branch $Y_{2}$ is iteratively refined using InceptionDWBlock, which employs multi-scale depthwise convolution kernels such as 3 × 3 and 5 × 5 in parallel to capture multi-scale spatial patterns. Specifically, each InceptionDWBlock internally splits its input along the channel dimension into two sub-groups processed by 3 × 3 and 5 × 5 depthwise convolutions, respectively, then concatenates and fuses the outputs through a 1 × 1 pointwise convolution with a residual connection to the block input. Two InceptionDWBlocks are cascaded to progressively extract hierarchical features:

(2) $$Y_{4}=\phi_{InceptDW}^{\left(1\right)}\left(Y_{2}\right),\;Y_{5}=\phi_{InceptDW}^{\left(2\right)}\left(Y_{4}\right)$$

where $\phi_{InceptDW}^{\left(i\right)}$ denotes the *i*-th InceptionDWBlock. This cascaded design enables the module to capture increasingly abstract multi-scale patterns while maintaining computational efficiency. The first block $\phi_{InceptDW}^{\left(1\right)}$ extracts initial multi-scale features from $Y_{2}$, producing $Y_{4}$$\in R^{C^{\prime }/4\times H\times W}$, while the second block $\phi_{InceptDW}^{\left(2\right)}$ further refines these features to generate $Y_{5}$$\in R^{C^{\prime }/4\times H\times W}$ with richer spatial representations. Through this two-stage cascaded processing, the effective receptive field progressively expands from 3 × 3/5 × 5 to 5 × 5/9 × 9, enabling the capture of spatial patterns ranging from fine textures to medium-range structures.

All branches are then aggregated through dense connections, and a 1 × 1 convolution fusion operation $\phi_{fusion}$ maps the concatenated multi-channel features back to the target dimension as follows:

(3) $$F_{out}=\phi_{fusion}\left(\mathrm{Concat}[Y_{0},Y_{1},Y_{2},Y_{3},Y_{4},Y_{5}]\right)$$

The concatenation operates along the channel dimension, yielding an intermediate tensor of $R^{(6\times C^{\prime }/4)\times H\times W}$, and $\phi_{fusion}$ reduces the channel count back to *C*, performing cross-branch feature interaction and compression simultaneously. This dense connection strategy ensures that features at different processing stages are all preserved in the final output. The original split features $Y_{2}$ and $Y_{3}$ provide low-level geometric information, while the refined features $Y_{4}$ and $Y_{5}$ contribute multi-scale spatial patterns learned through the InceptionDWBlocks. The fixed-scale branches $Y_{0}$ and $Y_{1}$ supply complementary channel-recombined and locally filtered representations, and this six-stream dense aggregation ensures maximum feature reuse across all processing stages.

**Figure 3:**
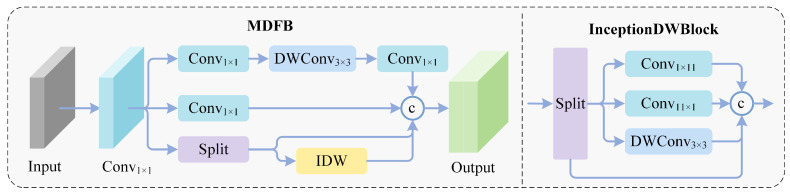
**Architecture of MDFB**. Input features are processed by ${\mathrm{Conv}}_{1\times 1}$, then split into three branches to concatenate. InceptionDWBlock with parallel ${\mathrm{Conv}}_{1\times 11}$, ${\mathrm{Conv}}_{11\times 1}$, and ${\mathrm{DWConv}}_{3\times 3}$ branches.

Compared to standard convolution blocks, as illustrated in Figure 3, MDFB reduces parameter count through feature splitting and depthwise convolutions while retaining rich multi-scale feature representations, ensuring that geometric details such as small object edge textures are not lost during forward propagation.

#### 3.2.2. Deep Layer Semantic Modeling Module

In deep-layer feature maps, the semantics of small objects have become relatively abstract, requiring a global receptive field to capture long-range context. The HAFMB module combines self-attention with multi-scale convolutions to achieve collaborative modeling of local and global features. Given input $X\in R^{C\times H\times W}$, a channel splitting strategy is adopted to divide it into a global branch $X_{g}\in R^{\alpha C\times H\times W}$ and a local branch $X_{l}\in R^{(1-\alpha )C\times H\times W}$, where α denotes the splitting ratio that controls the proportion of channels allocated to global self-attention modeling. We set α=0.5 by default, equally partitioning channels between the global and local branches. This balanced allocation ensures that sufficient channels are devoted to capturing long-range dependencies via self-attention, while the remaining channels preserve local detail information. The selection of α=0.5 is empirically validated through ablation experiments in Section 4.4.1, a channel splitting strategy is adopted to divide it into a global branch $X_{g}\in R^{0.5C\times H\times W}$ and a local branch $X_{l}\in R^{0.5C\times H\times W}$. In the global branch, spatial feature enhancement is performed through single-head self-attention as follows:

(4) $$X_{g}^{\prime }=\phi_{proj}\left(\mathrm{Softmax}\left(\frac{\phi_{Q}\left(X_{g}\right)\;\phi_{K}{\left(X_{g}\right)}^{T}}{\sqrt{d_{qk}}}\right)\phi_{V}\left(X_{g}\right)\right)$$

where $\phi_{Q}$, $\phi_{K}$, and $\phi_{V}$ are the query, key, and value mapping functions, respectively, and $d_{qk}$ is the dimension of query and key vectors. Multi-scale depthwise convolution branches are simultaneously introduced to enhance local representation, and final fusion is achieved through residual connections as follows:

(5) $$F_{out}=X+\phi_{final}\left(\mathrm{Concat}\left[X_{g}^{\prime },X_{l},\phi_{DW}^{3\times 3}\left(X\right),\phi_{DW}^{5\times 5}\left(X\right)\right]\right)$$

Through channel splitting and single-head attention design, global modeling capability is maintained while meeting lightweight requirements, providing semantically robust feature representations for the detection head.

### 3.3. High-Fidelity Feature Transformation Mechanism

Traditional methods employ nearest-neighbor or bilinear interpolation for upsampling, which cannot reconstruct sub-pixel level geometric details, leading to the degradation of small object edge sharpness and texture information. Meanwhile, simple concatenation of shallow and deep layer features ignores the essential differences in semantic hierarchy and spatial alignment, causing spatial misalignment during feature fusion. In the downsampling direction, when strided convolutions or max pooling directly reduce resolution, insufficient sampling rates trigger aliasing effects that convert high-frequency details into low-frequency noise. We propose a high-fidelity transformation framework covering the entire upsampling and downsampling process. The upsampling stage avoids interpolation blur through a channel cyclic shift mechanism, transforming resolution recovery into channel-spatial reorganization. The deep–shallow layer fusion stage resolves the semantic gap through bidirectional gating mechanisms, achieving adaptive alignment. The downsampling stage suppresses aliasing distortion through a three-branch parallel architecture.

#### 3.3.1. Channel-Adaptive Shift Upsampling

To address the inherent deficiencies of traditional upsampling, we reformulate the resolution recovery problem as channel-spatial reorganization. Given input $F_{\mathrm{in}}\in R^{C\times H\times W}$, local spatial patterns are first extracted through depthwise convolution as follows:

(6) $$F_{\mathrm{dw}}=\phi_{DW}^{3\times 3}\left(F_{\mathrm{in}}\right)$$

**Figure 4:**
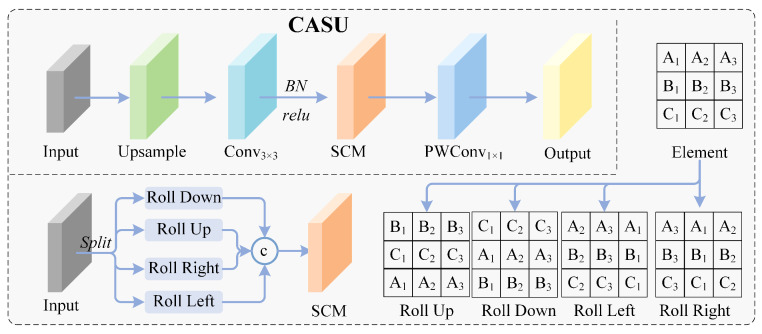
**Architecture of Channel-Adaptive Shift Upsampling (CASU).** Top is the main pipeline consisting of Upsample, ${\mathrm{Conv}}_{3\times 3}$ with BN and ReLU, Shift Channel Mixing (SCM), and ${\mathrm{PWConv}}_{1\times 1}$. Bottom is detailed structure of SCM, which splits input into four branches with Roll Down, Roll Up, Roll Right, and Roll Left operations, followed by concatenation. Right is visualization of the element shifting mechanism for each roll direction.

Subsequently, a channel shift mixing operation is introduced to cyclically shift feature channels along spatial dimensions, enabling adjacent positions to capture context from different receptive fields. For an upsampling factor of s=2, the shift operation is formalized as follows:

(7) $$F_{\mathrm{shift}}^{\left(i\right)}=\mathrm{CircShift}\left(F_{\mathrm{dw}}^{\left(i\right)},\delta_{i}\right),\;i\in \left\{1,\ldots ,C\right\}$$

where $\delta_{i}\in {\left\{-1,0,1\right\}}^{2}$ denotes the shift vector for the *i*-th channel. The shift direction is determined by a fixed cyclic assignment based on the channel index as follows: $\delta_{i}=D\left(i\;\mathrm{mod}\;4\right)$, where D={(0,−1),(0,1),(−1,0),(1,0)} corresponds to upward, downward, leftward, and rightward shifts, respectively. This deterministic pattern ensures that every four consecutive channels uniformly cover all cardinal directions, enabling the subsequent pointwise convolution to aggregate information from all four spatial neighborhoods for 2×2 sub-pixel reconstruction without introducing any learnable parameters.

(8) $$F_{\mathrm{up}}=\sigma \left(\phi_{pw}\left(F_{\mathrm{shift}}\right)\right)\in R^{C^{\prime }\times 2H\times 2W}$$

where $\phi_{pw}$ denotes pointwise convolution implemented as 1 × 1 convolution, and σ represents the activation function. This design effectively alleviates edge blur caused by traditional interpolation, providing a high-quality resolution foundation for subsequent deep–shallow layer feature fusion.The network are illustrated in Figure 4.

#### 3.3.2. Multi-Scale Context Alignment Fusion

Relying solely on high-fidelity upsampling is insufficient to bridge the semantic gap between deep and shallow layer features. Shallow layer features are rich in spatial details but have low semantic abstraction, while deep layer features possess rich semantics but limited resolution. The two exhibit significant distributional differences in feature space. We dynamically regulate fusion weights through bidirectional gating mechanisms to achieve adaptive balance between semantics and details.

Given the upsampled deep layer feature $F_{\mathrm{deep}}\in R^{C\times H\times W}$ and the shallow layer feature $F_{\mathrm{shallow}}\in R^{C\times H\times W}$ at the same resolution, adaptive gating weights are first generated through independent 1 × 1 convolutions as follows:

(9) $$G_{h}=\sigma \left(\phi_{1\times 1}^{h}\left(F_{\mathrm{shallow}}\right)\right),\;G_{l}=\sigma \left(\phi_{1\times 1}^{l}\left(F_{\mathrm{deep}}\right)\right)$$

where σ denotes the Sigmoid activation function, and $G_{h},G_{l}\in R^{C\times H\times W}$ control the fusion proportions of shallow and deep layer features, respectively, achieving pixel-wise adaptive weight allocation.

Based on the gating weights, bidirectional interaction enhancement is performed. Shallow layer features are refined through deep layer semantic guidance, while deep layer features recover local textures through shallow layer detail compensation:

(10) $$F_{h}^{\prime }=F_{\mathrm{shallow}}+G_{h}⊙F_{\mathrm{shallow}}+\left(1-G_{h}\right)⊙\left(G_{l}⊙F_{\mathrm{deep}}\right)$$

This formula achieves the self-enhancement of shallow layer features and the selective incorporation of deep layer semantics through the gating mechanism, where the term $\left(1-G_{h}\right)$ ensures complementary balance between the two types of information.

Finally, the bidirectionally enhanced features are aggregated through channel concatenation and convolution as follows:

(11) $$F_{\mathrm{fused}}=\phi_{conv}^{3\times 3}\left(\mathrm{Concat}(F_{h}^{\prime },F_{\mathrm{deep}})\right)$$

This operation concatenates the enhanced shallow layer features with the upsampled deep layer features along the channel dimension, learning the optimal fusion strategy through the convolutional layer. This enables the network to adaptively adjust the contributions of deep and shallow layer information according to object scale and scene complexity. For regions with dense small objects, the gating mechanism tends to preserve more shallow layer details, while in complex background regions, it relies on deep layer semantics for feature filtering.

#### 3.3.3. Frequency-Aware Downsampling (DRFD)

**Figure 5:**
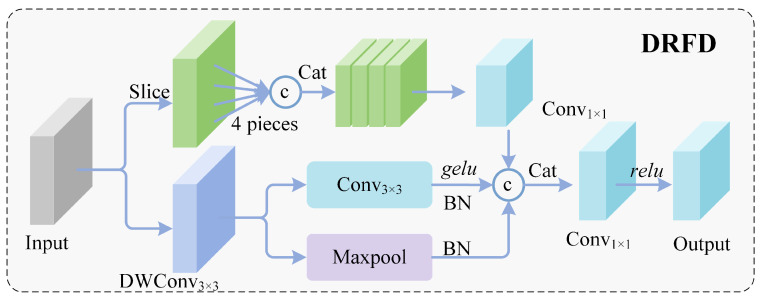
**Architecture of DRFD.** Input features are first processed by ${\mathrm{DWConv}}_{3\times 3}$, then split into two paths. Upper features are sliced into 4 pieces and concatenated, followed by ${\mathrm{Conv}}_{1\times 1}$. Lower parallel branches of ${\mathrm{Conv}}_{3\times 3}$ with BN and GELU, and Maxpool with BN, then concatenated. Both paths are merged via ${\mathrm{Conv}}_{1\times 1}$ with ReLU to produce the output.

Traditional strided convolutions or pooling operations act as low-pass filters. When the sampling rate is insufficient, high-frequency details such as small object edge textures undergo spectral aliasing, converting into unrecoverable noise. To address this problem, we adopt a three-branch parallel architecture, termed Diversified Residual Frequency-aware Downsampling (DRFD) Figure 5, to capture features from the following three dimensions: detail preservation, semantic extraction, and feature enhancement. The branches are fused through residual connections to minimize information loss.

Given input $X_{in}\in R^{C\times H\times W}$, the features are distributed into the following two main paths: a detail preservation path and a semantic-enhancement path, where the latter further consists of a convolution branch and a pooling branch, forming three parallel branches in total. The **detail preservation branch** employs the Cut operation, which performs Space-to-Depth transformation by periodically rearranging pixels from 2 × 2 neighborhoods into the channel dimension, achieving lossless geometric reorganization as follows:

(12) $$X_{cut}=\phi_{1\times 1}\left(\mathrm{Concat}(X_{00},X_{01},X_{10},X_{11})\right)$$

where $X_{ij}$ represents the sub-pixel grid sampled with stride 2 at position (i,j), with i,j∈{0,1}. This operation avoids high-frequency information loss inherent in traditional downsampling, completely preserving the original feature distribution.

The **semantic extraction branch** and **feature enhancement branch** extract abstract features and key object responses through strided convolution and max pooling, respectively, as follows:

(13) $$X_{conv}=\mathrm{ReLU}\left(\mathrm{BN}\left(\phi_{conv}^{3\times 3,s=2}\left(X_{in}\right)\right)\right),\;X_{max}=\mathrm{BN}\left({\mathrm{MaxPool}}_{2\times 2}\left(X_{in}\right)\right)$$

The semantic branch employs 3 × 3 convolution with stride 2 to learn contextual semantics, with outputs sequentially processed through batch normalization and GELU activation. The enhancement branch highlights local peak features through 2 × 2 max pooling, with outputs processed through batch normalization. The two branches collaborate in a complementary manner as follows: the convolution branch learns feature abstraction capability while the pooling branch enhances responses in object regions. Together they compensate for the insufficiency of the detail branch in semantic understanding.

After concatenating the three feature streams along the channel dimension, cross-channel interaction is performed through a convolutional layer with dimensionality reduction to the target channel count as follows:

(14) $$F_{down}=\phi_{fusion}^{1\times 1}\left(\mathrm{Concat}\left[X_{cut},X_{conv},X_{max}\right]\right)$$

This fusion strategy embodies the residual philosophy. The Cut branch preserves original detail information, while the convolution and pooling branches supplement semantic and enhancement features. The complementary mechanism among the three branches effectively suppresses downsampling aliasing effects, significantly improving the representation capability of deep layer features for small objects.

### 3.4. Multi-Scale Context Focus Aggregation

Although high-fidelity feature transformation ensures complete information transmission across hierarchical levels, the effective aggregation of multi-scale features still faces the fundamental challenge of limited receptive fields. The effective receptive field of networks is typically much smaller than the theoretical receptive field and concentrates in the central region of feature maps, resulting in the low utilization of edge information. For tiny objects that inherently lack discriminative texture features, long-range spatial context must be relied upon for inference.

**Figure 6:**
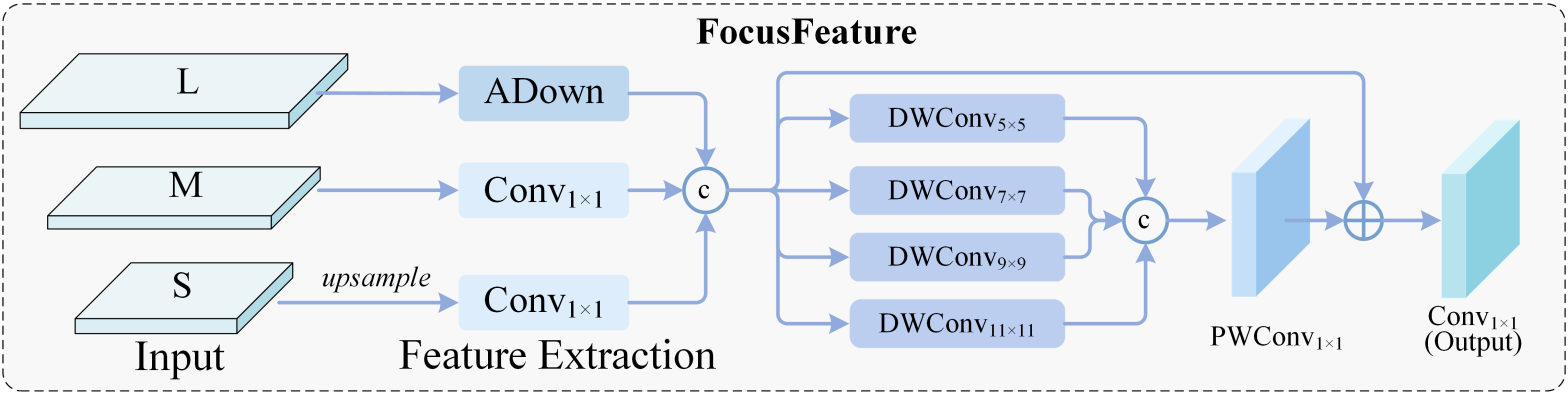
**Architecture of FocusFeature.** Multi-scale inputs (L, M, S) are processed through Feature Extraction—L via DRFD, M via ${\mathrm{Conv}}_{1\times 1}$, and S via CASU and ${\mathrm{Conv}}_{1\times 1}$—then concatenated. Then parallel multi-scale DWConv branches (${\mathrm{DWConv}}_{5\times 5}$, ${\mathrm{DWConv}}_{7\times 7}$, ${\mathrm{DWConv}}_{9\times 9}$, ${\mathrm{DWConv}}_{11\times 11}$), followed by concatenation, ${\mathrm{PWConv}}_{1\times 1}$, and ${\mathrm{Conv}}_{1\times 1}$ for output.

We design the Multi-Scale Contextual Focus Aggregation module, termed FocusFeature (Figure 6), inspired by the collaborative working mechanism of human foveal and peripheral vision. This module serves as the central hub of the feature pyramid, simultaneously receiving three feature streams from different processing stages as follows: $F_{P2}^{final}$ carrying high-resolution details, $F_{P3}^{fused}$ representing middle layer features, and $F_{P4}^{enc}$ encoding semantic abstraction. Multi-granularity spatial context focusing is achieved within a single module through parallel large-kernel depthwise convolutions.

First, spatial alignment is performed on the three inputs at different scales. To unify features to the P3 scale with 8× downsampling, frequency-aware downsampling using the three-branch architecture described in Equations (12)–(14) is applied to $F_{P2}^{final}$, Channel-Adaptive Shift Upsampling using the shift reorganization mechanism described in Equations (6)–(8) is applied to $F_{P4}^{enc}$, and $F_{P3}^{fused}$ undergoes direct channel alignment through 1 × 1 convolution. We concatenate the aligned features along the channel dimension, thereby obtaining a unified multi-scale representation $F_{\mathrm{align}}$ as follows:

(15) $$F_{\mathrm{align}}=\mathrm{Concat}\left(\mathrm{DownSample}\left(F_{P2}^{final}\right),\phi_{1\times 1}\left(F_{P3}^{fused}\right),\mathrm{UpSample}\left(F_{P4}^{enc}\right)\right)$$

where DownSample(·) and UpSample(·) denote the frequency-aware downsampling and Channel-Adaptive Shift Upsampling operations, respectively.

After obtaining the aligned features, a multi-granularity parallel focusing mechanism is introduced, where depthwise convolution kernels of different sizes K={5,7,9,11} simultaneously operate on $F_{\mathrm{align}}$. Small kernels capture local fine textures, corresponding to the high-resolution perception of foveal vision, while large kernels establish long-range spatial dependencies, simulating the wide-field background understanding of peripheral vision. The parallel multi-kernel outputs are fused with the original features through residual connections and undergo cross-channel interaction via pointwise convolution as follows:

(16) $$F_{P3}^{focus}=\phi_{pw}^{\left(1\right)}\left(F_{\mathrm{align}}+\phi_{pw}^{\left(2\right)}\left(\sum_{k\in K}\phi_{DW}^{k\times k}\left(F_{\mathrm{align}}\right)\right)\right)$$

Through the FocusFeature module, multi-granularity receptive field coverage ranging from 5 × 5 to 11 × 11 is achieved within a single aggregation layer. Compared to traditional FPN architectures that require stacking dozens of convolutional layers to achieve equivalent receptive fields, this design significantly improves computational efficiency. More importantly, this parallel large-kernel architecture enables the model to perceive deep-layer environmental semantic context through large kernels while preserving the shallow-layer geometric details of tiny objects, achieving cross-scale reasoning capability that discerns the significant from the subtle.

To accommodate the dual-scale input requirements of the decoder, $F_{P3}^{focus}$ directly serves as the enhanced P3 feature. Simultaneously, to generate semantically consistent deep-layer features, $F_{P3}^{focus}$ is fed into the frequency-aware downsampling module again for 2× downsampling as follows:

(17) $$F_{P4}^{focus}=\mathrm{DownSample}\left(F_{P3}^{focus}\right)$$

This design ensures that $F_{P3}^{focus}$ and $F_{P4}^{focus}$ are highly aligned in contextual semantics, while the three-branch architecture further suppresses aliasing noise when generating P4 features, maintaining semantic fidelity. Compared to traditional three-scale schemes, the dual-scale features significantly reduce decoder computational complexity while avoiding information loss of small objects.

### 3.5. Decoder and Training Strategy

To preserve the advantages of end-to-end training, we inherit the decoder architecture and training strategy from DEIM, while performing key adaptations for UAV small object detection. The deeply optimized dual-scale features $F_{P3}^{focus}$ at the middle layer with 8× downsampling and $F_{P4}^{focus}$ at the deep layer with 16× downsampling are fed into the D-FINE Transformer decoder. This decoder redefines the regression task as a distribution modeling problem and progressively refines prediction results on dual-scale feature maps through an IoU-aware query selection strategy.

The training phase employs Dense O2O matching strategy and Matchability-Aware Loss. Dense O2O strengthens supervision signals by increasing the number of target matches per image, addressing the sparse supervision problem inherent in traditional one-to-one matching. Matchability-Aware Loss performs adaptive optimization for matches of different quality levels, applying stronger supervision to high-quality matches while adopting more relaxed constraints for low-quality matches, significantly accelerating model convergence. This training strategy is particularly suitable for detecting dense small objects in UAV scenarios, improving training efficiency while ensuring accuracy.

Although P2 features are extracted by the backbone, they are deliberately excluded from the final decoding stage. This is because P2 at 160×160 resolution would incur quadratic attention complexity in the Transformer decoder, violating real-time constraints, while its semantically weak responses may introduce low-level noise that degrades query matching quality. Crucially, P2 information is not discarded but already absorbed into $F_{P3}^{focus}$ and $F_{P4}^{focus}$ through the upstream MCAF gated fusion and FocusFeature large-kernel aggregation, rendering direct P2 decoding both redundant and counterproductive.

Unlike DEIM, our decoder only performs predictions on dual-scale features consisting of P3 and P4, avoiding the loss of small object information caused by the deepest layer features. This design maintains the advantages of end-to-end training while achieving specialized optimization for UAV small object detection, ultimately generating high-confidence bounding boxes and category scores.

## 4. Experiments and Results

### 4.1. Datasets and Evaluation Metrics

#### 4.1.1. Dataset Setup

To comprehensively validate the effectiveness and robustness of HMF-DEIM in UAV perspectives, this study employs the following aerial imagery detection datasets for experiments: **VisDrone2019 Dataset.**

Within the global UAV vision field, this dataset has established itself as a foundational benchmark and a highly regarded resource. It encompasses complex imagery from 14 cities across China under varying weather conditions (cloudy and sunny), illumination settings (daytime and nighttime), and scene densities. The dataset contains 10 fine-grained object categories as follows: pedestrian, people, bicycle, car, van, truck, tricycle, awning-tricycle, bus, and motor. Following the official protocol, we partition the dataset into 6471 training images, 548 validation images, and 1610 test images. All images are uniformly resized to 640 × 640 resolution for training and testing.

To provide a detailed understanding of the test set composition, Table 1 reports the number of instances per category and per size group (small, medium, and large) in the VisDrone2019 test split, based on the official dataset statistics [55]. Small objects are defined as those with an area <$32^{2}$ pixels, medium objects with an area between $32^{2}$ and $96^{2}$ pixels, and large objects with an area >$96^{2}$ pixels.

#### 4.1.2. Evaluation Metrics

This study employs the standard COCO evaluation protocol and YOLO evaluation metrics for comprehensive assessment as follows:

1. **Average Precision (AP)**
  - **mAP^50^**: Average precision at IoU threshold of 0.5.
  - **mAP^50–95^**:Average precision across IoU thresholds from 0.5 to 0.95 with a step size of 0.05, providing a more rigorous reflection of high-precision localization capability.
2. **Efficiency Metrics**
  - **FPS (Frames Per Second)**: Real-time processing capability measured on NVIDIA A100-PCIE-40 GB.
  - **GFLOPs (Giga Floating-point Operations)**: Measuring model computational complexity.
  - **Params (Parameters)**: Model parameter count, evaluating storage requirements and deployment feasibility.

### 4.2. Implementation Details

#### 4.2.1. Training Strategy

To ensure fair comparison, the proposed HMF-DEIM model and the compared DEIM baseline are trained without loading any pretrained weights. All models adopt unified training configurations, with specific parameter settings shown in Table 2.

To ensure valid comparison, all baseline models are trained and tested under identical hardware environments and hyperparameter settings. The DEIM baseline adopts the exact same training configuration as HMF-DEIM.

#### 4.2.2. Evaluation Metrics

This study employs multiple key metrics to comprehensively evaluate model performance across different datasets, including Precision, Recall, F1 Score, mean Average Precision (mAP^50^, mAP^50–95^), inference speed (FPS), floating-point operations (FLOPs), and model parameters (Params).

The accuracy of object detection models relies on prediction correctness, evaluated through Intersection over Union (IoU) and thresholds ∈ [0, 1], specifically using True Positives (TPs), False Positives (FPs), and False Negatives (FNs) metrics as follows:

- **TP (True Positive)**: Correct predictions when IoU exceeds the specified threshold.
- **FP (False Positive)**: Incorrect predictions of non-existent objects, or detected objects with IoU below the threshold.
- **FN (False Negative)**: Objects present in the actual image that the model fails to predict.

It should be noted that True Negatives (TNs) are not used in object detection, as the number of bounding boxes that should not be predicted in each image is infinite.

Based on the above confusion matrix elements, the following evaluation metrics are defined:

**Precision** measures the proportion of correct predictions among all predicted bounding boxes as follows:

(18) $$\mathrm{Precision}=\frac{TP}{TP+FP}$$

**Recall** represents the proportion of objects correctly localized and identified among ground truth annotations as follows:

(19) $$\mathrm{Recall}=\frac{TP}{TP+FN}$$

**F1 Score** as the harmonic mean of precision and recall, provides a comprehensive evaluation of algorithm performance as follows:

(20) $$F1\text{-}\mathrm{score}=\frac{2PR}{P+R}$$

**Mean Average Precision (mAP)** is the primary metric for evaluating object detection model accuracy. **mAP^50^** represents the average precision when the IoU threshold is 0.5, expressed as the integral area under the Precision-Recall curve (P-R curve). **mAP^50–95^** considers average precision across IoU thresholds from 0.5 to 0.95 (step size 0.05), providing a more rigorous reflection of the model’s high-precision localization capability.

The mAP is calculated as follows:

(21) $$\mathrm{mAP}=\frac{1}{n}\sum_{i=1}^{n}AP_{i}$$

where *n* denotes the number of categories, and $AP_{i}$ represents the average precision for the *i*-th category. **The scale-specific metrics are as follows**:

- **AP_*s*_ (Small)**: Average precision for small objects (area < 32^2^).
- **AP_*m*_ (Medium)**: Average precision for medium objects (32^2^ < area < 96^2^).
- **AP_*l*_ (Large)**: Average precision for large objects (area > 96^2^).

Finally, the metrics for evaluating model complexity and inference speed are considered as follows:

1. **FPS** is defined as the number of frames processed by the model per second. Typically, based on specific application scenarios, real-time object detection models need to achieve a frame rate of no less than 40–50 FPS when handling video frames to fulfill practical task requirements.
2. **FLOPs** represent the number of floating-point operations required per second. This is a key metric for measuring the speed and computational capability of neural network models. Lower FLOPs indicate lower computational complexity.
3. **Model Parameters**, measured in millions (M), reflect both model scale and complexity, and are commonly used as critical indicators for evaluating lightweight architectures. In general, a smaller parameter count enables faster inference and facilitates deployment on edge devices with limited computational resources.

The comprehensive evaluation of the above metrics provides a thorough reflection of model performance in terms of detection accuracy, computational efficiency, and deployment feasibility.

### 4.3. Main Results Analysis

#### 4.3.1. Comprehensive Comparison on the VisDrone2019 Test Set

To evaluate HMF-DEIM’s performance, we compare it with 18 mainstream detection models on the VisDrone2019 test set, covering classic Transformer detectors, CNN detectors, latest YOLO series, and DEIM series models. Table 1 presents the detailed performance comparison results.

As shown in Table 3, HMF-DEIM demonstrates excellent comprehensive performance on the VisDrone2019 test set, achieving an mAP^50^ of **0.405** and an mAP^50–95^ of **0.235**. Compared to the baseline **DEIM-D-Fine-S**, HMF-DEIM achieves an absolute improvement of **2.1%** in mAP^50^ and **1.6%** in mAP^50–95^ with only a 1.69M increase in parameters. Most notably, on the **AP_*s*_** metric reflecting tiny object detection capability, HMF-DEIM achieves a relative improvement of up to **21.3%**. This strongly demonstrates that removing redundant deep-layer features (P5) and introducing the high-fidelity feature transformation mechanism can effectively address the small object information loss problem in UAV perspectives.

Compared to the SOTA model **D-Fine-S** at a similar scale, HMF-DEIM surpasses it by 1.1% in mAP^50^ and 2.0% in AP_*s*_, while also achieving significant advantages in large object AP_*l*_. Furthermore, compared to the classic Transformer model **DINO-R50** that employs high-resolution input (750, 1333), HMF-DEIM achieves comparable performance in AP_*s*_ using only (640, 640) input resolution, while requiring merely 25% of its parameters and 12.4% of its GFLOPs. This result indicates that targeted architectural optimization is more efficient than simply increasing input resolution or stacking parameters.

It is important to note that our core comparative experiments are conducted at the S (Small) scale to ensure fair comparison under the same parameter regime. To address potential concerns about environmental consistency, in this revision we have re-run D-Fine-M and DEIM-D-Fine-M under our unified experimental environment with identical data preprocessing, input resolution (640×640), augmentation protocols, evaluation scripts, and IoU threshold settings. The updated results in Table 3 confirm that D-Fine-M achieves a higher mAP^50^ (0.410) than HMF-DEIM (0.405), which is expected given its substantially larger model capacity (19.19 M vs. 11.87 M parameters). However, HMF-DEIM significantly outperforms D-Fine-M in the most critical metric for UAV scenarios—AP_*s*_ (0.148 vs. 0.133, a relative improvement of 11.3%)—and also achieves a higher AP_*l*_ (0.489 vs. 0.479). This result demonstrates that our architectural innovations specifically benefit small object detection, which is the primary focus of this work. Compared to DEIM-D-Fine-M (19.19 M params), HMF-DEIM achieves superior performance across nearly all metrics with only 61.9% of the parameters, validating the effectiveness of our hierarchical differentiation and high-fidelity transformation design. All evaluation metrics follow the standard COCO evaluation protocol, as adopted in DEIM [23].

Regarding statistical significance, we conduct bootstrap resampling (10,000 iterations) on the VisDrone2019 test set to compute 95% confidence intervals for the key metrics. Specifically, we resample the 1610 test images with replacement, recompute mAP^50^ and AP_*s*_ for both HMF-DEIM and the baseline DEIM-D-Fine-S on each bootstrap sample, and report the 2.5th and 97.5th percentiles. The resulting 95% confidence intervals are as follows: HMF-DEIM mAP^50^: [0.399,0.411] vs. DEIM-D-Fine-S mAP^50^: [0.378,0.390]; HMF-DEIM AP_*s*_: [0.141,0.155] vs. DEIM-D-Fine-S AP_*s*_: [0.115,0.129]. The non-overlapping confidence intervals confirm the statistical significance of the improvements (p<0.05). Moreover, the per-class analysis in Table 4 shows that HMF-DEIM achieves consistent positive AP improvements across all 10 categories. The gains are independently corroborated on two additional datasets with markedly different scale distributions and imaging conditions as follows: AI-TOD-v2 (28,036 images, 700,621 instances, +6.2% mAP^50^) and DOTA-v1.5 (+5.4% mAP^50^). The convergence of bootstrap-verified confidence intervals, consistent per-class improvements, and cross-dataset validation collectively establishes the robustness and statistical reliability of the reported performance gains.

In terms of computational efficiency, although the GFLOPs of HMF-DEIM increase by approximately 37% compared to the baseline DEIM-D-Fine-S, this is primarily attributed to the multi-scale large-kernel convolutions in the FocusFeature module and the full-chain high-fidelity transformation computations. Nevertheless, benefiting from the dual-scale decoding strategy that reduces partial detection head overhead, the model achieves a TensorRT FP16 inference speed of **465 FPS** on NVIDIA A100 GPU. This demonstrates that HMF-DEIM maintains extremely high inference efficiency while significantly improving detection accuracy, capable of meeting the stringent requirements for real-time processing on UAV onboard platforms.

#### 4.3.2. Fine-Grained Category Performance Analysis

Table 4 presents the detailed performance of HMF-DEIM across the 10 categories in the VisDrone2019 dataset, with comprehensive comparison against the baseline model DEIM-D-Fine-S. Experimental results demonstrate that HMF-DEIM achieves performance improvements across all categories, with an average AP improvement of 7.3%. Among these, the improvement magnitudes for objects of different scales exhibit distinctly differentiated characteristics, directly reflecting the effectiveness of the architectural improvement strategies proposed in this paper.

On small object categories, HMF-DEIM exhibits the most significant performance improvements. Specifically, the AP improvements for pedestrian, people, bicycle, and motor categories all exceed 7.6%, with the pedestrian category achieving the highest relative improvement of 11.7%. This result directly validates the rationale for removing the deepest layer—for these small objects, the feature map dimensions at the deepest layer have already decreased to below one pixel, completely unable to preserve effective spatial information, instead introducing noise and increasing computational burden. Meanwhile, the high-fidelity feature fusion mechanism significantly enhances the model’s representation capability for small objects by preserving more original detail information from the P3 layer. It is noteworthy that objects in the 28–36 pixel size range achieve 8–11% improvement magnitudes; these objects are precisely at the critical point of visibility disappearance after 32× downsampling, making the removal of the P5 layer particularly impactful for them.

For medium and large object categories, although the baseline model has already achieved relatively high detection accuracy on these objects, HMF-DEIM still achieves stable improvements of 4–6%. This indicates that the dual-scale detection strategy and hierarchical multi-scale feature fusion mechanism proposed in this paper are not solely optimized for small objects, but they provide universal gains for objects across all scales. Additionally, on category pairs with highly similar visual appearances, the performance improvement of HMF-DEIM is more pronounced. This benefits from the contextual semantic information introduced by the MCAF module and the multi-scale focusing capability of FocusFeature, enabling the model to perform more precise category discrimination through surrounding environmental features and fine-grained local patterns, rather than relying solely on the shape and texture features of the objects themselves.

### 4.4. Ablation Experiments

To systematically validate the effectiveness of each component in HMF-DEIM, we conduct progressive ablation experiments on the VisDrone2019 test set. As shown in Table 5, using DEIM-D-Fine-S as the baseline, each module demonstrates a clear cumulative gain effect.

The ablation analysis proceeds in a hierarchical manner to isolate the contribution of each architectural component. **Removal of the redundant deepest layer P5** constitutes the first modification. By constraining the detection scales to P3-P4, the model achieves a 5.7% improvement in AP_*s*_ while simultaneously reducing computational cost by 13.7%. This counterintuitive result strongly validates our hypothesis as follows: in tiny object detection from UAV perspectives, the P5 layer with 32× downsampling not only loses target information but also introduces background noise. **Introduction of the hierarchical differentiation backbone network** represents the subsequent enhancement. Specifically, the shallow P2 features are enabled in the backbone, and the MDFB and HAFMB modules are applied at their respective hierarchical levels. Although incorporating high-resolution P2 features causes computational cost to slightly increase to 25.2 GFLOPs, this overhead remains within a controllable range due to the lightweight design of depthwise separable convolutions. Concurrently, AP_*s*_ significantly improves to 0.135, indicating that preserving the geometric details of P2 in the feature extraction stage is crucial for small object representation. **The high-fidelity feature transformation modules**—comprising CASU, MCAF, and DRFD—are progressively integrated to ensure the integrity of P2-P4 feature flow during cross-scale interactions. Notably, the MCAF module further improves AP_*s*_ by utilizing fine-grained information from P2 for semantic alignment during the feature fusion stage. Although processing the P2 layer incurs additional computational growth, this investment is justified by the steady improvements in detection accuracy. **The FocusFeature module**, serving as the core aggregation node, completes the architectural integration. This module aligns multi-scale features containing P2 information and performs large-kernel aggregation, ultimately outputting enhanced P3/P4 features for decoding. The complete HMF-DEIM model achieves mAP^50^ = 0.405 and AP_*s*_ = 0.148. Although the final computational cost increases by approximately 37% compared to the baseline, the AP_*s*_ metric demonstrates a substantial relative improvement of 21.3%, while the model maintains extremely high inference speed. This computational investment thus offers exceptional cost-effectiveness, demonstrating that HMF-DEIM achieves an optimal balance between leveraging P2 information for feature enhancement and maintaining efficient prediction through dual-scale (P3/P4) decoding.

#### 4.4.1. Channel Splitting Ratio in HAFMB

The HAFMB module employs a channel splitting strategy to partition input features into a global branch (processed by single-head self-attention) and a local branch (identity mapping). The splitting ratio α determines the proportion of channels allocated to global modeling. To identify the optimal configuration, we conduct ablation experiments on the VisDrone2019 test set by varying α∈{0.25,0.5,0.75}, with all other settings kept identical to the full HMF-DEIM model.

As shown in Table 6, the splitting ratio α=0.5 achieves the best overall performance. When α=0.25, the limited number of channels allocated to self-attention restricts the module’s global modeling capability, resulting in a 0.6% drop in mAP^50^ and a notable decline in AP_*s*_. Conversely, when α=0.75, although more channels participate in global attention, the reduced local branch weakens the preservation of fine-grained spatial details, leading to marginal performance degradation with increased parameters. The balanced α=0.5 configuration provides the optimal trade-off between global semantic modeling and local detail preservation, which is consistent with the design principle that deep-layer features require both long-range contextual understanding and retention of discriminative local patterns.

#### 4.4.2. Comparison of Upsampling Methods

To evaluate the effectiveness of the proposed CASU module, we compare it against representative upsampling methods by replacing all upsampling operators in HMF-DEIM while keeping other components unchanged. The compared methods include Nearest-neighbor interpolation, Bilinear interpolation, CARAFE [38] (a content-aware reassembly operator), and DySample [56] (a dynamic upsampling method). All experiments are conducted on the VisDrone2019 test set under identical training settings.

As shown in Table 7, conventional interpolation methods (Nearest and Bilinear) yield the lowest accuracy due to their inability to reconstruct sub-pixel details, particularly for tiny objects (AP_*s*_ of 0.136 and 0.138). Content-aware methods CARAFE and DySample improve accuracy by learning adaptive upsampling kernels, but thye introduce additional learnable parameters (+0.48 M and +0.31 M, respectively) and higher computational cost. In contrast, CASU achieves the best accuracy across all metrics while maintaining lower parameters and GFLOPs than both CARAFE and DySample. This efficiency advantage stems from CASU’s parameter-free cyclic shift mechanism, which rearranges existing channel information for sub-pixel reconstruction rather than learning content-dependent kernels, achieving an effective trade-off between reconstruction quality and computational overhead.

### 4.5. Generalization Experiments

To validate the robustness of the HMF-DEIM architecture and its generalization capability under different aerial imaging conditions, we conduct evaluations on two additional highly challenging datasets, **AI-TOD-v2** and **DOTA-v1.5**.

#### 4.5.1. AI-TOD-v2 Tiny Object Detection

**The AI-TOD-v2 dataset** is specifically designed for tiny object detection, containing 28,036 images and 700,621 instances, with an average object size of only 12.8 pixels, and 86% of objects smaller than 16 pixels. The dataset is officially split into training (11,214 images), validation (5607 images), and test (11,215 images) sets. Since AI-TOD-v2 provides complete training, validation, and test set partitions with corresponding annotations, we conduct evaluations on both the validation and test sets. In the experiments, we maintain hyperparameter settings consistent with VisDrone2019, with only necessary adjustments made according to computational resources.

As shown in Table 8, experiments on AI-TOD-v2 reveal that HMF-DEIM possesses solid generalization ability. On the test set, our method achieves **0.586 mAP^50^** and **0.248 mAP^50–95^**, representing improvements of **6.2%** and **2.7%** over the baseline, respectively. This indicates that our architecture successfully recovers a substantial number of extremely tiny objects that are missed by traditional methods through preserving shallow features (P2) and high-fidelity feature transmission. The consistent improvements on both validation and test sets demonstrate the stability and reliability of our method.To provide broader context, we additionally compare with YOLOv11-S (representing the YOLO paradigm) and D-Fine-S (representing the DETR paradigm). On the test set, HMF-DEIM surpasses D-Fine-S by 5.8% and YOLOv11-S by 16.8% in mAP^50^, confirming the generalization advantage of our architecture on datasets dominated by extremely tiny objects.

#### 4.5.2. DOTA-v1.5 Cross-Domain and Cross-Scale Generalization Experiments

**The DOTA-v1.5 dataset** provides large-scale, multi-category aerial images, containing 403,318 instances across 16 categories. The dataset comprises 2806 images, with official splits of 1411 training images, 458 validation images, and 937 test images. Since the official test set labels are not publicly available, all comparative experiments are conducted on the validation set. The experiments adopt a batch size of 2, training for 300 epochs, with other hyperparameters kept consistent with the VisDrone2019 experimental configuration. As shown in Table 9, We crop high-resolution images into 1024 × 1024 sub-images for processing (HBB mode).

The experimental results demonstrate that HMF-DEIM achieves **0.712 mAP^50^** and **0.462 mAP^50–95^** on DOTA-v1.5, representing improvements of **5.4%** and **4.4%** over the baseline, respectively. The F1 score improvement reaches **3.8%**. This result validates that the FocusFeature module effectively expands the receptive field through parallel large-kernel convolutions, enabling the model to achieve contextual understanding capability for large-scale objects at shallower network layers (P3/P4), while the CASU upsampling module renders the boundaries of densely arranged small objects more distinct. Compared to D-Fine-S and YOLOv11-S, HMF-DEIM achieves improvements of 6.3% and 13.6% in mAP^50^, respectively, demonstrating consistent superiority across both DETR-based and YOLO-based paradigms in diverse aerial scenarios.

### 4.6. Visualization

To intuitively demonstrate the detection performance of HMF-DEIM and the functional mechanisms of each module, this section conducts visualization studies from the following two dimensions: detection result comparison and feature response analysis. All visualization results are generated based on typical samples from the VisDrone2019 test set and AI-TOD-v2 dataset.

**Figure 7:**
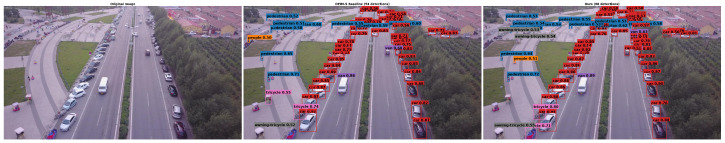
Dense small object scenario.

**Figure 8:**
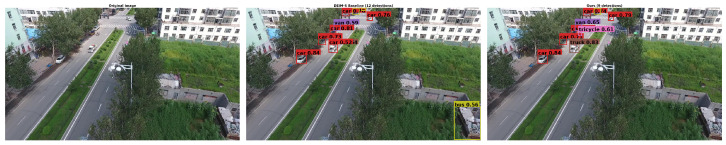
Complex background scenario.

**Figure 9:**
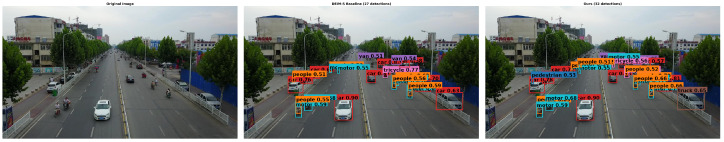
Extreme scale variation scenario.

Figure 7, Figure 8 and Figure 9 present detection result comparisons in three typical scenarios. Each figure displays, from left to right, the original image, DEIM-D-Fine-S (baseline) detection results, and HMF-DEIM (ours) detection results.

In the dense small object scenario illustrated in Figure 7, the baseline model detects 54 objects but exhibits obvious missed detections for smaller pedestrians and motorcycles in the upper half of the image. In contrast, HMF-DEIM detects 14 additional objects in the same scenario with generally higher detection confidence. This improvement benefits from two design aspects as follows: removing the P5 layer prevents complete information loss of small objects after 32× downsampling, and the FocusFeature module enhances the feature discriminability of small objects through cross-scale context aggregation.

Figure 8 demonstrates the complex background scenario, where semantic confusion in deep features causes the baseline model to misidentify some building edges as vehicle objects, such as misrecognizing building structures in the lower right corner as buses, while also exhibiting missed detections for objects with colors similar to buildings. HMF-DEIM effectively suppresses background noise interference through global semantic modeling in the HAFMB, eliminates the aforementioned false detections, and successfully detects occluded or low-contrast vehicle objects.

The extreme scale variation scenario shown in Figure 9 reveals that while the baseline model can accurately detect nearby large vehicles, it almost completely misses tiny motorcycles at the far end of the road. HMF-DEIM detects five additional tiny objects in this scenario, achieving balanced detection across all scales. This result validates the effectiveness of the CASU-MCAF-DRFD full-chain high-fidelity feature transformation framework, where CASU avoids edge blur during upsampling, MCAF bridges the semantic gap between deep and shallow layer features, and DRFD suppresses aliasing effects during downsampling. The three components work synergistically to ensure the integrity of small object features during cross-scale transfer.

#### 4.6.1. Feature Response Heatmap Analysis

**Figure 10:**
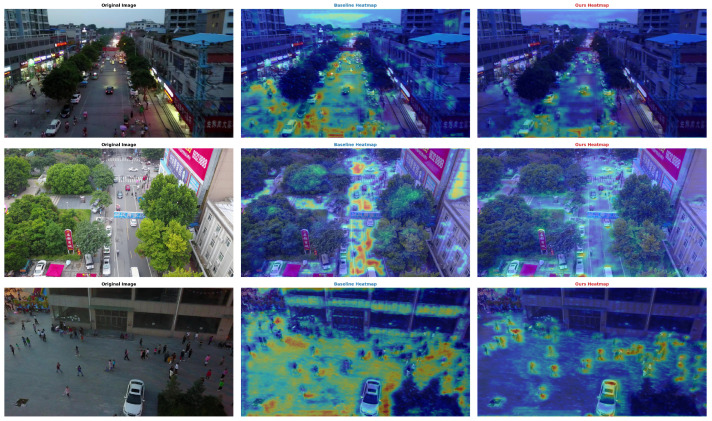
**Feature heatmap comparison in tiny object scenarios**. (**Left**) Original image (red boxes marking dense small object regions). (**Middle**) DEIM-D-Fine-S heatmap. (**Right**) HMF-DEIM heatmap. Darker colors in the heatmap indicate higher response intensity of the model to that region.

To further validate the model’s perception capability for tiny objects, Figure 10 presents a comparison of feature response heatmaps on the AI-TOD-v2 dataset. This dataset has an average object size of only 12.8 pixels, with 86% of objects smaller than 16 pixels, posing extremely high requirements on the detail perception capability of detection models.

Comparing the heatmaps of the two models reveals several notable differences. In terms of response intensity, the baseline model’s heatmap shows relatively low response intensity in object regions, with diffuse response ranges and blurred boundaries with background regions, indicating that the model struggles to effectively capture discriminative features of tiny objects. The HMF-DEIM heatmap, by contrast, exhibits stronger and more concentrated responses in object regions with clearly discernible object contours, benefiting from the MDFB module’s high-fidelity preservation of shallow-to-middle layer geometric details.

Regarding background suppression, the baseline model’s heatmap exhibits local over-response phenomena where excessively dark colors in object center regions lead to feature saturation, while background regions show scattered weak response noise. HMF-DEIM achieves precise focusing on object regions through the HAFMB’s channel splitting strategy and single-head self-attention mechanism, effectively suppressing background noise. Quantitative statistics further demonstrate that the baseline model’s detection rate for tiny objects in this scenario is less than 50%, with a large number of pedestrian and non-motorized vehicle objects being missed. HMF-DEIM improves the detection rate to over 70% through the FocusFeature module’s multi-scale large-kernel convolution aggregation, with higher IoU alignment between detection boxes and actual object positions.

#### 4.6.2. Visualization Conclusions

Based on the comprehensive visualization analysis above, several important conclusions emerge. HMF-DEIM significantly outperforms the baseline model in detection performance across UAV aerial scenarios including dense small objects, complex backgrounds, and extreme scale variations, with particularly notable improvements in the detection rate and localization accuracy for tiny objects. The synergistic action of the hierarchically differentiated backbone network combining shallow-to-middle layer MDFB with deep layer HAFMB, the high-fidelity feature transformation framework CASU-MCAF-DRFD, and the cross-scale focus aggregation module FocusFeature enable the model to effectively capture fine-grained features of small objects, suppress complex background noise, and establish cross-scale contextual associations. The visualization results intuitively validate the design intent of each core module as follows: removing the P5 layer solves the problem of complete small object information loss, CASU eliminates interpolation blur, DRFD suppresses aliasing effects, and FocusFeature expands the effective receptive field, collectively providing reliable guarantees for the model’s high-precision detection.

## 5. Conclusions

This paper presents HMF-DEIM, a novel end-to-end detection architecture specifically designed to address the challenges of small object detection in UAV aerial scenarios. Based on comprehensive analyses and validations, we confirm that fusing hierarchical differential feature extraction, full-link high-fidelity feature transformation, and cross-scale contextual focus aggregation can effectively boost detection performance under multi-scale conditions and complex aerial environments.

HMF-DEIM achieves significant performance gains across multiple benchmark datasets. On the VisDrone2019 test set, it improves mAP^50^ by 2.1% and mAP^50–95^ by 1.6% compared to the baseline, with a remarkable relative improvement of 21.3% in AP_*s*_ for tiny object detection. On the AI-TOD-v2 test set, the model demonstrates increases of 6.2% in mAP^50^ and 2.7% in mAP^50–95^, while on the DOTA-v1.5 validation set, improvements of 5.4% in mAP^50^ and 4.4% in mAP^50–95^ are achieved. Ablation studies further confirm the synergistic effectiveness of each architectural component as follows: removing the redundant P5 layer yields a 5.7% improvement in AP_*s*_ while reducing computational cost, and the progressive integration of MDFB, HAFMB, and the high-fidelity transformation modules demonstrates clear cumulative gains. Notably, HMF-DEIM achieves a TensorRT FP16 inference speed of 465FPS on an NVIDIA A100 GPU, fully meeting the stringent real-time processing requirements of UAV onboard platforms. The overall experimental findings verify that the model performs effectively in complicated real-world UAV environments and exhibits great promise for real applications.

Although the proposed method achieves notable gains, several aspects warrant further investigation. First, the multi-scale large-kernel convolutions in the FocusFeature module, while effective for expanding the receptive field, increase GFLOPs by approximately 37% compared to the baseline; future work will explore decomposed convolution strategies and alternative feature aggregation schemes to retain cross-scale modeling capacity while cutting computational costs. Second, objects under severe occlusion in dense urban scenarios remain challenging; occlusion-aware learning mechanisms capable of reconstructing holistic object features from partial visibility will be investigated, with particular emphasis on discriminating between visually confusable categories such as pedestrians and people. Third, objects of extremely small scale (below 8×8 pixels) continue to pose difficulties despite our improved architecture, as evidenced by the AI-TOD-v2 experiments in which 86% of targets measure under 16 pixels; improved shallow-layer feature representations and purpose-built attention mechanisms for sub-pixel objects will be pursued. Beyond these, adaptive multi-resolution processing pipelines will be explored to efficiently manage ultra-high-resolution aerial imagery on resource-limited UAV systems, and hardware-aware compression strategies—namely quantization and pruning—will be adopted to facilitate efficient deployment on UAV onboard processors, reconciling computational efficiency with detection performance by leveraging dedicated neural processing hardware.

## Figures and Tables

**Table 1:** Instance distribution of VisDrone2019 test set by category and size.

Category	Total Instances	Small	Medium	Large
pedestrian	21,006	18,848	2066	92
people	6376	6024	349	3
bicycle	1302	1066	231	5
car	28,074	15,112	11,851	1111
van	5771	2906	2675	190
truck	2659	777	1445	437
tricycle	530	263	249	18
awning-tricycle	599	274	300	25
bus	2940	702	1715	523
motor	5845	4844	995	6
**Total**	**75,102**	**50,816**	**21,876**	**2410**

**Table 2:** Model training configuration parameters.

Parameter Category	Parameter Name	Setting Value
**Optimization Settings**	Optimizer	AdamW
	Initial Learning Rate	$4\times 10^{-4}$
	Weight Decay	$1\times 10^{-4}$
	Learning Rate Schedule	Cosine Annealing
	Warmup Iterations	2000
**Training Parameters**	Training Epochs	300 Epochs
	Batch Size	4
	Input Size	640×640
**Data Augmentation**	Early Augmentation (First 280 Epochs)	Mosaic
	Late Augmentation (Last 20 Epochs)	Standard Augmentation

**Table 3:** Comprehensive comparison of different object detection models on the VisDrone2019 test set.

Model	Input Size	mAP^50^	mAP^50–95^	AP_*S*_	AP_*M*_	AP_*L*_	Params (M)	GFLOPs
RT-DETR-R18	(640, 640)	0.363	0.208	0.113	0.305	0.413	19.89	57.0
DINO-R50	(750, 1333)	0.445	0.253	0.150	0.371	0.503	47.56	274.0
Faster-RCNN-R50-FPN	(768, 1344)	0.329	0.194	0.095	0.309	0.429	41.39	208.0
ATSS-R50-FPN-DyHead	(768, 1344)	0.338	0.204	0.100	0.317	0.485	38.91	110.0
YOLOv8-S	(640, 640)	0.307	0.173	0.078	0.269	0.372	11.13	28.5
YOLOv8-M	(640, 640)	0.332	0.190	0.090	0.294	0.417	25.85	78.7
YOLOv10-S	(640, 640)	0.323	0.179	0.086	0.278	0.361	7.22	21.4
YOLOv10-M	(640, 640)	0.345	0.195	0.097	0.300	0.414	15.32	58.9
YOLOv11-S	(640, 640)	0.313	0.176	0.080	0.272	0.364	9.42	21.3
YOLOv11-M	(640, 640)	0.350	0.203	0.098	0.312	0.413	20.04	67.7
YOLOv12-M	(640, 640)	0.336	0.192	0.094	0.298	0.386	20.11	67.2
RTMDet-Tiny	(640, 640)	0.312	0.184	0.077	0.288	0.445	4.88	8.0
FBRT-YOLO-M	(640, 640)	0.344	0.196	0.094	0.309	0.421	7.36	58.7
D-Fine-S	(640, 640)	0.394	0.227	0.128	0.331	0.468	10.18	24.9
D-Fine-M	(640, 640)	0.410	0.238	0.133	0.349	0.479	19.19	56.4
DEIM-D-Fine-S (Baseline)	(640, 640)	0.384	0.219	0.122	0.321	0.397	10.18	24.9
DEIM-D-Fine-M	(640, 640)	0.403	0.233	0.131	0.339	0.450	19.19	56.4
DEIMV2-S	(640, 640)	0.363	0.204	0.109	0.299	0.451	9.67	25.4
**HMF-DEIM (Ours)**	**(640, 640)**	**0.405**	**0.235**	**0.148**	**0.346**	**0.489**	**11.87**	**34.1**

**Table 4:** Detailed performance comparison by category on VisDrone2019 (COCO metrics).

Category	Avg. Size	DEIM-D-Fine-S (Baseline)			HMF-DEIM (Ours)			Improvement
	(Pixels)	AP	AP^50^	AP^75^	AP	AP^50^	AP^75^	ΔAP
pedestrian	22 × 48	0.145	0.362	0.095	**0.162**	**0.385**	**0.107**	+11.7%
people	18 × 42	0.095	0.271	0.045	**0.106**	**0.290**	**0.050**	+11.6%
bicycle	28 × 36	0.071	0.165	0.046	**0.079**	**0.178**	**0.051**	+11.3%
car	45 × 68	0.498	0.779	0.561	**0.522**	**0.798**	**0.583**	+4.8%
van	52 × 78	0.295	0.440	0.343	**0.313**	**0.456**	**0.361**	+6.1%
truck	68 × 96	0.251	0.399	0.281	**0.267**	**0.415**	**0.297**	+6.4%
tricycle	32 × 48	0.131	0.249	0.140	**0.142**	**0.263**	**0.148**	+8.4%
awning-tricycle	36 × 52	0.125	0.215	0.147	**0.136**	**0.228**	**0.156**	+8.8%
bus	88 × 124	0.420	0.595	0.481	**0.438**	**0.612**	**0.497**	+4.3%
motor	26 × 42	0.172	0.405	0.116	**0.185**	**0.423**	**0.124**	+7.6%
**Average**	**-**	**0.219**	**0.384**	**0.226**	**0.235**	**0.405**	**0.237**	**+7.3%**

**Table 5:** Component ablation experiment results (VisDrone2019 test set).

No.	Model Configuration	Backbone	Scale Config (Feature/Head)	MDFB	HAFMB	CASU	MCAF	DRFD	FF	mAP^50^	AP_*s*_	Params (M)	GFLOPs	ΔParams (M)	ΔGFLOPs
1	**Baseline**	HGNetv2	Feat: P3-P5/Head: P3-P5	–	–	–	–	–	–	0.384	0.122	10.18	24.9	–	–
2	+ Remove P5	HGNetv2	**Feat: P3-P4/Head: P3-P4**	–	–	–	–	–	–	0.389	0.129	9.84	21.5	−0.34	−3.4
3	+ Custom Backbone	**Custom**	**Feat: P2-P4/Head: P3-P4**	✓	✓	–	–	–	–	0.393	0.135	10.21	25.2	+0.37	+3.7
4	+ CASU	Custom	Feat: P2-P4/Head: P3-P4	✓	✓	✓	–	–	–	0.397	0.140	10.48	26.7	+0.27	+1.5
5	+ MCAF	Custom	Feat: P2-P4/Head: P3-P4	✓	✓	✓	✓	–	–	0.400	0.144	10.89	29.2	+0.41	+2.5
6	+ DRFD	Custom	Feat: P2-P4/Head: P3-P4	✓	✓	✓	✓	✓	–	0.403	0.146	11.24	31.2	+0.35	+2.0
7	**HMF-DEIM**	Custom	**Feat: P2-P4/Head: P3-P4**	✓	✓	✓	✓	✓	✓	**0.405**	**0.148**	**11.87**	**34.1**	**+0.63**	**+2.9**

**Table 6:** Ablation study on channel splitting ratio α in HAFMB (VisDrone2019 test set).

Splitting Ratio α	Global/Local	mAP^50^	mAP^50–95^	AP_*s*_	Params (M)
0.25	25%/75%	0.399	0.230	0.141	11.52
**0.5 (default)**	**50%/50%**	**0.405**	**0.235**	**0.148**	**11.87**
0.75	75%/25%	0.402	0.233	0.146	12.23

**Table 7:** Comparison of different upsampling methods in HMF-DEIM (VisDrone2019 test set).

Upsampling Method	mAP^50^	mAP^50–95^	AP*_s_*	Params(M)	GFLOPs
Nearest	0.393	0.226	0.136	11.62	32.8
Bilinear	0.395	0.228	0.138	11.62	32.9
CARAFE [38]	0.401	0.232	0.144	12.35	36.4
DySample [56]	0.403	0.234	0.146	12.18	35.7
**CASU (Ours)**	**0.405**	**0.235**	**0.148**	**11.87**	**34.1**

**Table 8:** Performance comparison on the AI-TOD-v2 dataset.

Model	P_Val	R_Val	${\mathrm{mAP}}_{50}^{\mathrm{Val}}$	${\mathrm{mAP}}_{50:95}^{\mathrm{Val}}$	F1_Val	P_Test	R_Test	${\mathrm{mAP}}_{50}^{\mathrm{Test}}$	${\mathrm{mAP}}_{50:95}^{\mathrm{Test}}$	F1_Test
YOLOv11-S	0.594	0.448	0.438	0.172	0.511	0.586	0.431	0.418	0.162	0.497
D-Fine-S	0.652	0.533	0.545	0.235	0.587	0.645	0.518	0.528	0.224	0.575
DEIM-D-Fine-S (Baseline)	0.658	0.542	0.551	0.238	0.594	0.651	0.528	0.524	0.221	0.583
**HMF-DEIM (Ours)**	**0.689**	**0.618**	**0.612**	**0.267**	**0.652**	**0.682**	**0.603**	**0.586**	**0.248**	**0.640**

**Table 9:** Performance comparison on the DOTA-v1.5 validation set (HBB mode).

Model	P_Val	R_Val	${\mathrm{mAP}}_{50}^{\mathrm{Val}}$	${\mathrm{mAP}}_{50:95}^{\mathrm{Val}}$	F1_Val
YOLOv11-S	0.632	0.565	0.576	0.352	0.597
D-Fine-S	0.688	0.631	0.649	0.412	0.658
DEIM-D-Fine-S (Baseline)	0.694	0.638	0.658	0.418	0.665
**HMF-DEIM (Ours)**	**0.718**	**0.689**	**0.712**	**0.462**	**0.703**

## Data Availability

Data sharing is not applicable to this article.

## Abbreviations

The following abbreviations are used in this manuscript:

UAV	Unmanned aerial vehicle
MDFB	Multi-domain feature blending
HAFMB	Hierarchical attention-guided feature modulation block
CASU	Channel-adaptive shift upsampling
MCAF	Multi-scale context alignment fusion
DRFD	Diversified residual frequency-aware downsampling
DETR	Detection transformer
FPN	Feature pyramid network
mAP	Mean average precision
FPS	Frames per second
FLOPs	Floating-point operations per second
TP	True positive
FP	False positive
FN	False negative
TN	True negative
IoU	Intersection over union
